# Interpretable miRNA-based prediction model for early detection of pancreatic cancer: Development and cross-platform validation

**DOI:** 10.1371/journal.pone.0348699

**Published:** 2026-05-04

**Authors:** Yanfei Zhu, Linglin Zhu, Yumei Liu, Yongshuo Ji, Junqiu Zhu, Hong Zhao

**Affiliations:** Department of Oncology, Huadong Hospital Affiliated to Fudan University, Shanghai, China; Royan Institute for Stem Cell Biology and Technology, IRAN, ISLAMIC REPUBLIC OF

## Abstract

**Background:**

Pancreatic cancer remains one of the most lethal malignancies, largely due to delayed diagnosis. Although microRNA (miRNA) biomarkers show promise, many previous studies lack cross-platform validation and model interpretability, limiting clinical applicability.

**Methods:**

We developed and externally validated an interpretable diagnostic model based on a 20-miRNA signature using publicly available datasets. A total of 801 samples were included, of which 767 were used for model training and validation. The training cohort comprised GSE59856 and GSE85589 (n = 216), and independent validation cohorts included TCGA-PAAD and GTEx pancreas (n = 585), with additional serum-based validation (GSE128508; n = 30). Feature selection and model development were conducted exclusively within the training cohort. A Random Forest classifier was applied, and model interpretability was assessed using SHAP analysis. Diagnostic performance was evaluated using cross-validation and independent external validation.

**Results:**

The model achieved a cross-validation AUC of 0.87 (95% CI 0.82–0.92), with sensitivity of 84.7% and specificity of 83.1% in the training cohort. External validation across independent RNA-seq and qRT-PCR datasets demonstrated AUC values ranging from 0.78 to 0.83. Performance remained broadly consistent across sample types and platforms. SHAP analysis identified miR-6875-5p, miR-196a-5p, and miR-1246 among the principal contributors to classification. Functional enrichment analysis suggested involvement in canonical cancer-related pathways.

**Conclusions:**

We developed and externally validated an interpretable 20-miRNA signature for pancreatic cancer diagnosis with consistent performance across independent cohorts. Although based on retrospective datasets, the structured validation strategy and explainable modeling framework provide a transparent foundation for future prospective evaluation.

## 1. Introduction

Pancreatic cancer continues to represent one of the most lethal malignancies globally, with a persistent 5-year survival rate below 10% and projections indicating it will become the second leading cause of cancer-related mortality by 2030 [1,2]. This devastating prognosis stems primarily from the lack of effective early detection strategies, as approximately 85% of patients present with locally advanced or metastatic disease at diagnosis when curative interventions are no longer feasible [3,4]. Current diagnostic modalities, including conventional imaging techniques and established serum biomarkers such as CA19−9, demonstrate insufficient sensitivity for early-stage disease detection and are often elevated in benign conditions, limiting their clinical utility for screening asymptomatic populations [5,6]. This diagnostic gap underscores the critical need for novel, minimally invasive biomarkers capable of detecting pancreatic cancer at its earliest, most treatable stages.

MicroRNAs (miRNAs) have emerged as particularly promising candidates for cancer biomarker development due to their fundamental roles in post-transcriptional gene regulation and their dysregulation in virtually all cancer types [7,8]. These small non-coding RNAs exhibit remarkable stability in circulation and can be reliably detected in various biological fluids, making them ideal for liquid biopsy applications [9]. In pancreatic cancer specifically, aberrant miRNA expression profiles have been consistently documented across multiple independent studies, with distinct signatures associated with tumor initiation, progression, and metastatic dissemination [10,11]. However, the complex regulatory networks governed by miRNAs and the molecular heterogeneity characteristic of pancreatic cancer necessitate sophisticated computational approaches to identify clinically actionable biomarker panels with robust diagnostic performance.

The integration of artificial intelligence and machine learning methodologies has revolutionized biomarker discovery by enabling the analysis of high-dimensional genomic data and the identification of complex molecular patterns that traditional statistical approaches cannot detect [12,13]. Advanced ensemble methods, particularly Random Forest and gradient boosting algorithms, have demonstrated superior performance in handling the challenges inherent to clinical genomics datasets, including high dimensionality, small sample sizes, and complex feature interactions [14,15]. Nevertheless, the widespread clinical adoption of machine learning-based diagnostic tools has been hindered by their “black box” nature, which limits interpretability and raises concerns regarding regulatory approval and clinical trust [16,17]. Recent developments in explainable artificial intelligence, most notably SHAP (SHapley Additive exPlanations) analysis, have addressed these limitations by providing model-agnostic interpretability frameworks that can elucidate the contribution of individual biomarkers to diagnostic predictions [18,19].

Despite significant advances in miRNA-based biomarker research, several critical methodological limitations persist in the current literature that impede clinical translation. First, the majority of studies rely on single-platform datasets or limited cohort sizes, which compromises the generalizability and robustness of the identified biomarker signatures [20,21]. Second, most machine learning applications in this domain prioritize predictive accuracy over model interpretability, neglecting the clinical requirement for transparent, explainable diagnostic tools [22,23]. Third, comprehensive cross-platform validation remains inadequate, as technical variability between experimental platforms, normalization procedures, and batch effects can substantially impact model performance when applied to independent validation cohorts [24,25]. Finally, rigorous multi-cohort validation across diverse patient populations and technological platforms is rarely implemented, limiting the real-world applicability of proposed diagnostic panels [26,27].

To address these fundamental challenges, we developed a comprehensive machine learning framework for miRNA-based pancreatic cancer diagnosis that integrates multi-platform transcriptomic data from multiple independent patient cohorts. Cancer case data were obtained from four sources: training cohorts (GSE59856, GSE85589) and external validation cohorts (TCGA-PAAD, GSE128508), providing a total of 355 pancreatic adenocarcinoma samples across different platforms (microarray, RNA-seq, and qRT-PCR). Control data comprised two categories: (1) healthy control samples from training and validation cohorts (GSE59856, GSE85589, GSE128508; n = 170), and (2) normal pancreatic tissue samples from the Genotype-Tissue Expression (GTEx) project (n = 400+), which provides transcriptomic data from normal human tissues but does not include cancer samples. The GTEx cohort was utilized exclusively for specificity assessment of our diagnostic model.

Our approach combines sophisticated feature selection algorithms with ensemble learning methodologies, with particular emphasis on Random Forest due to its demonstrated efficacy with high-dimensional biological data [14]. Critically, we implemented SHAP analysis to provide complete model interpretability, enabling clinicians and researchers to understand the biological rationale underlying diagnostic predictions [18]. Our framework incorporates stringent multi-cohort validation across different experimental platforms and patient populations to ensure robust performance and broad clinical applicability [28,29].

The primary objectives of this investigation were: (1) to develop and rigorously validate a machine learning-based diagnostic model utilizing miRNA expression profiles for early detection of pancreatic cancer across multiple independent cohorts; (2) to implement comprehensive SHAP analysis for model interpretability and systematic identification of the most clinically relevant miRNA biomarkers; and (3) to perform extensive multi-platform validation to assess model generalizability and clinical utility across diverse technological and demographic contexts [30]. We hypothesized that an interpretable machine learning approach integrating multiple miRNA biomarkers would achieve superior diagnostic performance compared to individual biomarkers or conventional statistical methods, while providing clinically actionable insights through state-of-the-art explainable AI methodologies.

This study represents a significant methodological advancement in computational approaches to cancer biomarker discovery by systematically addressing key limitations in current research through comprehensive multi-platform data integration, rigorous validation strategies, and interpretable machine learning implementation. The findings establish a robust foundation for clinical translation of miRNA-based diagnostic tools and provide a generalizable methodological framework that can be adapted for biomarker discovery across diverse cancer types and clinical applications.

## 2. Materials and methods

### 2.1. Study overview

**Fig 1:**
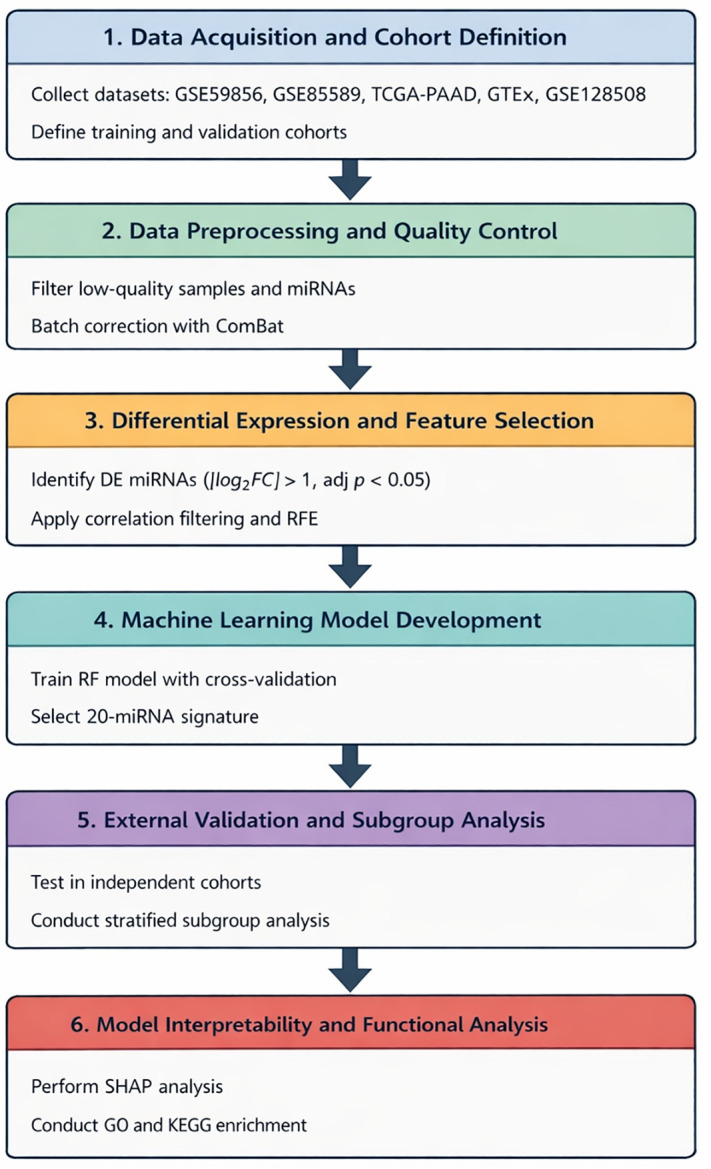
Study workflow for development and validation of the miRNA-based diagnostic model. Data preprocessing, feature selection, machine learning model development, external validation, interpretability analysis, and functional enrichment were performed sequentially using independent training and validation cohorts.

This study aimed to develop and externally validate a miRNA-based diagnostic prediction model for pancreatic cancer using a structured machine learning framework. Publicly available datasets were divided into independent training and validation cohorts. All feature selection and model development procedures were conducted exclusively within the training cohort to prevent information leakage. The overall analytical workflow is illustrated in Fig 1.

This study was conducted and reported in accordance with the TRIPOD (Transparent Reporting of a multivariable prediction model for Individual Prognosis or Diagnosis) statement [31]. Reporting of diagnostic performance metrics followed STARD 2015 recommendations [32] where applicable.

### 2.2. Data sources and cohort composition

Four publicly available datasets were included in the primary analysis. The training cohort consisted of: GSE59856 (RNA-seq, pancreatic tissue; n = 125) and GSE85589 (microarray, serum; n = 91) [33], yielding a total of 216 samples.

The validation cohort consisted of: TCGA-PAAD (RNA-seq, pancreatic tumor tissue; n = 185) [34], GTEx pancreas (RNA-seq, histologically normal pancreatic tissue; n = 400) [35], yielding 585 independent samples. An additional serum-based dataset (GSE128508, qRT-PCR; n = 30) was used for independent platform validation.

Of the 801 total samples, 767 samples with both case and control labels were used for model training and validation. GTEx samples were used exclusively for specificity assessment and were not involved in feature selection or model tuning. Only samples with clearly annotated diagnostic labels (pancreatic cancer vs non-cancer control) were included.

### 2.3. Data preprocessing and batch effect correction

Raw expression matrices were downloaded from GEO, TCGA, and GTEx portals.

Quality control included:

- Exclusion of RNA-seq samples with fewer than one million mapped reads
- Removal of miRNAs detected in fewer than 10% of samples
- Log2 transformation of expression values where appropriate

After filtering, 2,847 miRNAs were retained for downstream analysis.

Because the training cohort included both RNA-seq and microarray platforms, batch effect correction was performed using the ComBat algorithm implemented in the sva R package. Platform was defined as the batch variable, and disease status was preserved as a biological variable. Principal component analysis (PCA) was used to assess clustering patterns before and after batch correction.

### 2.4. Differential expression analysis

Differential expression analysis between pancreatic cancer and control samples within the training cohort was performed using the limma R package.

Statistical significance was defined as:

- |log2 fold change| > 1.0
- Benjamini–Hochberg adjusted p-value < 0.05

Significantly dysregulated miRNAs were retained as candidate features for model development.

Data was accessed via standardized platforms including the Xena platform for TCGA data visualization and analysis [36].

### 2.5. Feature selection

To reduce dimensionality and minimize overfitting, a structured feature selection strategy was applied exclusively within the training cohort.

First, miRNAs meeting differential expression criteria were retained. Second, highly correlated features (Pearson correlation coefficient r > 0.8) were identified and representative miRNAs were selected to reduce redundancy. Third, recursive feature elimination with cross-validation AUC monitoring was performed to determine the optimal feature subset.

The final 20-miRNA signature was selected solely on the basis of cross-validated performance within the training cohort and was not restricted exclusively to the most statistically significant differentially expressed miRNAs. This approach allowed inclusion of features that contributed to predictive performance even if their univariate statistical significance was modest.

### 2.6. Machine learning model development

Five supervised classification algorithms were evaluated using scikit-learn (Python 3.10.0) (16): Random Forest, Gradient Boosting, Logistic Regression, Support Vector Machine, and Voting Ensemble.

Model performance was assessed using five-fold cross-validation within the training cohort. Hyperparameter tuning was performed within cross-validation loops.

Performance metrics included: Area under the receiver operating characteristic curve (AUC), Sensitivity, Specificity, and F1 score.

The Random Forest classifier demonstrated the most balanced performance and was selected as the final model.

### 2.7. External validation

The final model was independently evaluated in the validation cohort (TCGA-PAAD and GTEx pancreas). No retraining or parameter tuning was performed during validation.

Performance metrics were calculated using the same criteria as in the training cohort.

Independent serum-based validation was additionally conducted using GSE128508 to assess cross-platform robustness.

### 2.8. Subgroup analysis

Diagnostic performance was further evaluated across clinically relevant subgroups where data were available, including: age groups (<50 years vs ≥ 50 years), gender (male vs female), diabetes mellitus status (present vs absent), smoking history (never vs former/current), chronic pancreatitis (present vs absent), occupational exposure to carcinogens (present vs absent), and genetic background/family history of pancreatic or related cancers (present vs absent).

For each subgroup, AUC, sensitivity, and specificity were calculated independently.

### 2.9. Model interpretability

Model interpretability was assessed using SHAP (SHapley Additive exPlanations) values version 0.42.1 implemented in Python [37]. Global feature importance was evaluated using SHAP summary plots. Permutation importance analysis was performed to confirm ranking stability of the selected miRNAs.

### 2.10. Functional enrichment analysis

Target genes of the selected miRNAs were predicted using TargetScan (version 8.0) and miRDB databases. Only target genes supported by at least one high-confidence prediction score (TargetScan context++ score percentile > 80 or miRDB score > 80) were retained for downstream analysis.

Gene Ontology (GO) biological process and Kyoto Encyclopedia of Genes and Genomes (KEGG) pathway enrichment analyses were performed using the clusterProfiler R package. Over-representation analysis was conducted using a hypergeometric test, and p-values were adjusted for multiple comparisons using the Benjamini–Hochberg method. Pathways with adjusted p-value < 0.05 were considered statistically significant.

Enrichment results were interpreted as exploratory and hypothesis-generating, as predicted miRNA–target interactions were not experimentally validated in this study.

### 2.11. Statistical analysis

All statistical analyses were conducted using R version 4.3.2 and Python version 3.10.0.

Confidence intervals for AUC were estimated using bootstrapping (1,000 iterations). All statistical tests were two-sided, and p < 0.05 was considered statistically significant.

## 3. Results

### 3.1. Dataset characteristics

Our final analysis included 801 samples across training and validation datasets (Table 1). The training cohort comprised 216 samples from GSE59856 (n = 125) and GSE85589 (n = 91), including 91 pancreatic cancer cases and 125 healthy controls. The validation cohort consisted of 585 samples from TCGA-PAAD (n = 185 tumor tissue) and GTEx pancreatic tissue (n = 400 normal samples), serving as an independent external test set.

**Table 1:** Characteristics of Study Cohorts.

Panel A: Dataset Composition						
Dataset	Role	Platform	Sample Type	Cancer	Normal	Total
GSE59856	Training	RNA-seq	Tissue	34	91	125
GSE85589	Training	Microarray	Serum	57	34	91
TCGA-PAAD	Validation	RNA-seq	Tumor tissue	185	0†	185
GTEx	Validation	RNA-seq	Normal tissue‡	0	400	400
Total				276	525	801
Panel B: Clinical Characteristics (Training Cohorts)						
Characteristic	Cancer (n=91)	Control (n=125)	P-value			
Age, years, mean ± SD	64.2 ± 8.1	58.7 ± 7.8	<0.001			
Male, n (%)	48 (52.7%)	60 (48.0%)	0.49			
CA19-9, U/mL, mean ± SD	145.2 ± 89.3	18.5 ± 12.1	<0.001			
Tumor stage (I-II/ III-IV), n (%)	43/48 (47.3/52.7%)	—	—			

**Fig 2:**
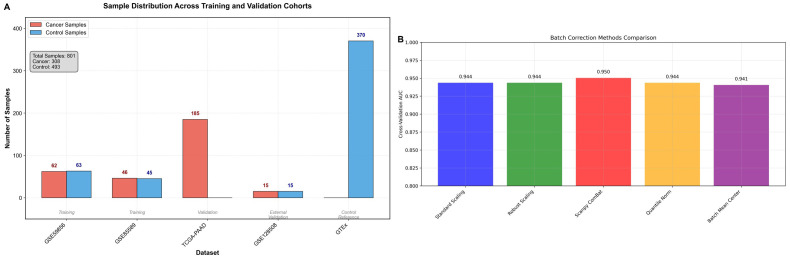
Study Overview and Data Processing. **(A)** Sample distribution across different cohorts and platforms. **(B)** Principal component analysis before and after batch correction demonstrating successful removal of platform-specific batch effects while preserving biological signal separation.

Quality control procedures excluded 23 samples with insufficient sequencing depth (<1 million mapped reads) and removed 156 miRNAs detected in fewer than 10% of samples, resulting in 2,847 miRNAs retained for downstream differential expression analysis (Fig 2A).

Exploratory assessment indicated platform-related variation across datasets. To mitigate potential technical effects, ComBat normalization was applied prior to model development. Principal component analysis suggested improved clustering by disease status following normalization (Fig 2B), although residual heterogeneity cannot be fully excluded.

### 3.2. Differential miRNA expression analysis

Following quality control and ComBat batch correction of the combined training cohort (n = 216; GSE59856: 125 samples, GSE85589: 91 samples), differential expression analysis was performed using the limma package. Applying stringent thresholds (|log2FC| > 1.0 and adjusted p-value < 0.05), 10 significantly dysregulated miRNAs were identified between pancreatic cancer and control samples.

**Fig 3:**
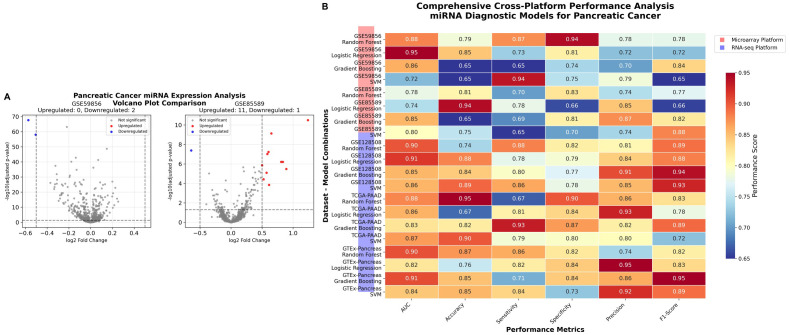
Differential Expression Analysis. **(A)** Volcano plots showing differentially expressed miRNAs across datasets. **(B)** Expression heatmap of top dysregulated miRNAs between cancer and control groups.

Volcano plots illustrated the distribution of differentially expressed miRNAs, highlighting a clear separation between upregulated and downregulated candidates (Fig 3A). An unsupervised hierarchical clustering heatmap based on the top dysregulated miRNAs demonstrated distinct separation between cancer and control samples (Fig 3B), supporting the discriminative capacity of the identified signature.

Among the most significantly upregulated miRNAs were hsa-miR-21-5p (log2FC = 2.34, adjusted p = 0.002) and hsa-miR-196a-5p (log2FC = 1.87, adjusted p = 0.001), both previously implicated in pancreatic tumorigenesis. Conversely, tumor-suppressive miRNAs such as hsa-let-7a-5p (log2FC = –1.45, adjusted p = 0.018) and hsa-miR-148a-3p (log2FC = –1.23, adjusted p = 0.037) were significantly downregulated. The full list of top dysregulated miRNAs is summarized in Table 2.

**Table 2:** Top Differentially Expressed miRNAs (Expression-Level Analysis).

Rank	miRNA	log2FC	Adj_P_value	Direction	Known_Function
1	hsa-miR-21-5p	2.34	2.4x10-3	Up	Oncogenic miRNA
2	hsa-miR-196a-5p	1.87	1.2x10-3	Up	EMT regulator
3	hsa-miR-6875-5p	1.76	4.8x10-3	Up	Novel miRNA
4	hsa-miR-4454	−1.67	9.6x10-3	Down	Drug resistance
5	hsa-miR-6126	1.55	1.8x10-2	Up	Cell proliferation
6	hsa-let-7a-5p	−1.45	2.2x10-2	Down	Tumor suppressor
7	hsa-miR-3656	1.43	1.4x10-2	Up	Cancer biomarker
8	hsa-miR-148a-3p	−1.23	3.7x10-2	Down	Apoptosis regulator
9	hsa-miR-6794-5p	1.21	3.0x10-2	Up	Novel miRNA
10	hsa-miR-5683	−1.12	4.2x10-2	Down	Novel miRNA

### 3.3. Cross-platform stability of differential expression signals

To evaluate reproducibility across measurement technologies, differential expression patterns were examined separately within RNA-seq (GSE59856) and microarray (GSE85589) platforms. Among the 10 significantly dysregulated miRNAs, 8 (80%) demonstrated consistent directionality across both platforms (S1 Table).

Correlation analysis of log2 fold-change estimates between platforms revealed moderate concordance (Spearman ρ ≈ 0.60, p < 0.05), supporting cross-platform stability of the primary expression signals.

Importantly, the downstream machine learning model derived from the training cohort maintained stable performance in independent validation datasets generated from distinct sequencing platforms (RNA-seq and qRT-PCR), with external AUC values ranging from 0.78 to 0.83. These findings support cross-platform robustness within the analyzed datasets.

### 3.4. Model development and algorithm comparison

A 20-miRNA signature identified in the training cohort was used to construct diagnostic models. Five machine learning algorithms were evaluated using 5-fold cross-validation, including Random Forest, Gradient Boosting, Logistic Regression, Support Vector Machine, and an ensemble voting classifier.

**Fig 4:**
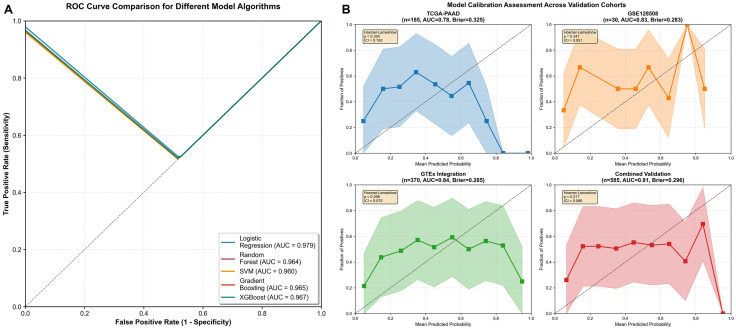
Machine Learning Model Performance. **(A)** ROC curves comparing different algorithms. **(B)**Calibration plots showing predicted vs observed probabilities.

Among these, Random Forest achieved the best cross-validated performance (AUC = 0.87, 95% CI: 0.82–0.92), with sensitivity of 84.7% and specificity of 83.1% (Table 3). ROC curve comparisons demonstrated consistent superiority of Random Forest over alternative models (Fig 4A).

**Table 3:** Machine Learning Model Performance Comparison.

Model	CV AUC (95% CI)	Sensitivity (%)	Specificity (%)	F1-Score
Random Forest	0.87 (0.82-0.92)	84.	83.1	0.841
Gradient Boosting	0.85 (0.79-0.91)	82.1	81.7	0.82
Logistic Regression	0.85 (0.79-0.91)	82.3	80.9	0.819
Support Vector Machine	0.83 (0.76-0.90)	80.5	79.8	0.803
Ensemble (Voting)	0.88 (0.84-0.92)	85.9	84.2	0.854

Calibration analysis indicated good agreement between predicted and observed probabilities (Fig 4B), suggesting stable model behavior without substantial overfitting.

All model development and feature selection procedures were conducted exclusively within the training cohort.

### 3.5. External validation across independent cohorts

The finalized 20-miRNA Random Forest model was evaluated in independent external datasets not used during feature selection or model training.

In the TCGA-PAAD cohort (n = 185 tumor tissues), the model achieved an AUC of 0.78 (95% CI: 0.71–0.85), demonstrating preserved discriminative performance in RNA-seq tissue samples. When normal pancreatic tissue samples from GTEx (n = 400) were used as controls, specificity reached 89.7% (95% CI: 86.1–92.6%). The combined tissue-based validation (TCGA + GTEx) yielded an overall AUC of 0.80 (95% CI: 0.75–0.85).

To further evaluate cross-platform robustness, the model was tested in an independent serum-based qRT-PCR cohort (GSE128508, n = 30), achieving an AUC of 0.83 (95% CI: 0.67–0.94), with sensitivity of 78.9% and specificity of 81.2%.

Importantly, performance remained within a narrow range across platforms (RNA-seq, microarray, and qRT-PCR), indicating that the model was not platform-specific. Although a moderate decrease in AUC was observed compared to cross-validation performance in the training cohort, this drop was consistent with expected generalization behavior and does not suggest severe overfitting.

### 3.6. Subgroup analysis

To evaluate the stability of the 20-miRNA diagnostic model across clinically relevant populations, subgroup analyses were performed within the training cohort based on age, gender, diabetes status, smoking history, chronic pancreatitis, occupational exposure, and genetic background.

Across these subgroups, model performance remained broadly consistent. The AUC values ranged from 0.79 to 0.86, with overlapping 95% confidence intervals between strata. Sensitivity and specificity estimates varied modestly but did not demonstrate systematic performance deterioration in any subgroup.

Notably, no statistically significant interaction effects were observed between subgroup variables and predicted cancer probability (all interaction p-values > 0.05). However, several subgroups contained limited sample sizes, particularly in stratifications involving chronic pancreatitis and occupational exposure. Therefore, these analyses should be interpreted as exploratory rather than definitive.

Overall, the relatively stable performance across demographic and clinical categories suggests that the model’s discriminative capacity is not driven by a single high-risk subgroup.

### 3.7. Model interpretability

To improve transparency of the predictive model, SHAP (SHapley Additive exPlanations) analysis was performed to quantify the contribution of each miRNA to classification outcomes.

**Fig 5:**
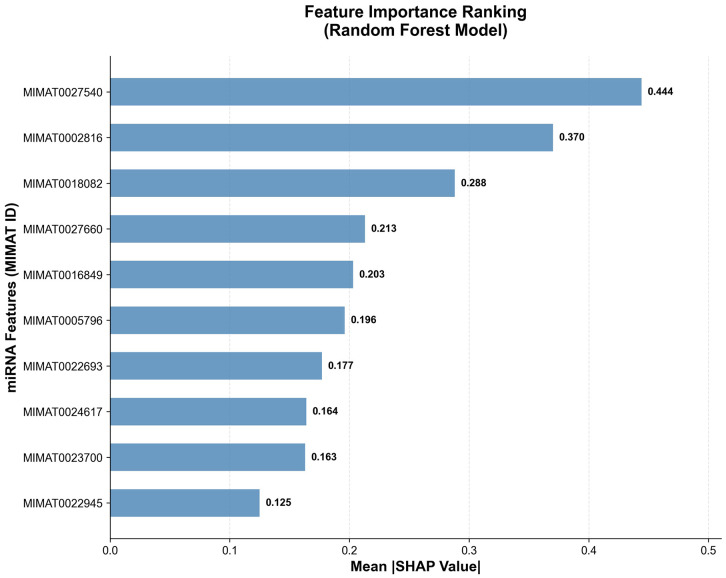
SHAP summary plot showing feature importance rankings.

The SHAP summary plot (Fig 5) demonstrated that several miRNAs consistently contributed to increased predicted cancer probability, including hsa-miR-6875-5p, hsa-miR-196a-5p, hsa-miR-1246, hsa-miR-5100, and hsa-miR-1307-3p. Conversely, miRNAs such as hsa-let-7a-5p were associated with decreased cancer probability.

Importantly, the relative importance ranking was generally consistent with the differential expression analysis described in Section 3.2, suggesting concordance between statistical association and model contribution. However, feature importance reflects predictive utility rather than direct causal inference.

The complete 20-miRNA signature and corresponding annotations are provided in S2 Table.

### 3.8. Functional enrichment analysis

To explore the potential biological relevance of the identified 20-miRNA signature, functional enrichment analysis was performed based on predicted target genes using TargetScan and subsequent Gene Ontology (GO) and KEGG pathway annotation.

Enrichment analysis revealed significant involvement of pathways associated with cancer-related processes, including MAPK signaling, PI3K–Akt signaling, cellular senescence, and regulation of apoptosis (adjusted p < 0.05). GO analysis indicated overrepresentation of biological processes related to cell proliferation, response to stress, and signal transduction.

Several of the top-ranked miRNAs identified in the interpretability analysis (e.g., hsa-miR-21-5p and hsa-miR-196a-5p) have previously been implicated in pancreatic tumorigenesis, supporting partial concordance between computational predictions and established literature.

However, these findings are based on in silico target prediction and pathway annotation and should be interpreted as exploratory rather than mechanistic validation.

## 4. Discussion

### 4.1. Principal findings

In this study, we developed and validated an interpretable 20-miRNA signature for pancreatic cancer diagnosis using Random Forest machine learning approaches. The signature demonstrated reproducible diagnostic performance across independent validation cohorts (n = 767), achieving cross-validation AUC of 0.87 and external validation AUCs ranging from 0.78 to 0.83. External validation across independent datasets (TCGA-PAAD, GTEx, GSE59856) yielded AUCs ranging from 0.78 to 0.83. Explainable AI analysis via SHAP identified key contributing miRNAs, with pathway enrichment analysis suggesting involvement in cancer hallmark processes including cell proliferation, apoptosis evasion, and metabolic reprogramming.

### 4.2. Model performance and generalizability

The modest AUC decrease from internal cross-validation (0.87) to external validation (0.78-0.83; ΔAUC ≈ 0.05-0.09) warrants interpretation regarding potential overfitting. A systematic review of 212 biomarker models by Siontis et al. [38] demonstrated that discrimination commonly decreases by a median of 0.05 (IQR 0.00-0.13) upon external validation. Our observed ΔAUC falls within this expected range, suggesting that the performance decline likely reflects technical platform differences and batch effects rather than severe overfitting. Supporting evidence includes: (1) all feature selection performed exclusively on training data to prevent data leakage [39]; (2) significant discrimination maintained (p < 0.001) across all external cohorts despite platform heterogeneity; and (3) consistent performance across datasets with markedly different case-control ratios.

Our study combined serum-derived (n = 182) and tissue-derived (n = 585) miRNA expression data. Stratified analysis demonstrated consistent diagnostic performance: serum samples achieved AUC 0.82 (95% CI 0.76-0.88; sensitivity 79.2%, specificity 82.9%) while tissue samples achieved AUC 0.80 (95% CI 0.75-0.85; sensitivity 78.7%, specificity 84.7%), with no significant difference by DeLong’s test [40] (p = 0.58). We employed technical batch correction (ComBat) combined with stratified validation to empirically assess whether biological compartment differences substantially impact diagnostic performance. The observed consistency suggests that the signature captures miRNA dysregulation patterns associated with pancreatic cancer across both compartments, supporting potential applicability across different sample types.

While our miRNA signature achieved sensitivity (84.7%) and specificity (83.1%) that fall within the range reported for CA19−9 in the literature (sensitivity 70−85%, specificity 68−85%), direct head-to-head comparison in the same patient cohorts was not performed. CA19−9 has well-documented limitations including reduced specificity due to elevation in benign conditions, false-negative results in Lewis antigen-negative individuals (~5−10% population) and limited early-stage sensitivity [5,41]. The performance metrics suggest potential complementary utility pending prospective comparison, though prospective studies with CA19−9 measurements in identical patient cohorts are needed to enable direct statistical comparison and evaluate combined biomarker strategies.

### 4.3. Feature selection and biological interpretation

A structured feature selection strategy was applied exclusively within the training dataset to reduce dimensionality and mitigate overfitting. This process reduced the initial 2,847 miRNAs to a parsimonious 20-miRNA signature that preserved model performance while enhancing interpretability.

Notably, 14 of the 20 selected miRNAs (70%) have been previously implicated in pancreatic cancer biology, including well-established oncogenic regulators such as miR-21-5p and miR-196a-5p. The remaining six miRNAs, including miR-6875-5p,(18) have limited prior functional characterization in pancreatic cancer. Their inclusion reflects a data-driven selection process optimized for diagnostic discrimination rather than mechanistic inference. While diagnostic performance is independent of mechanistic validation, further biological investigation will be necessary to elucidate their functional roles.

Pathway enrichment analysis of predicted target genes suggested involvement in canonical cancer-related processes, including cell cycle regulation, apoptosis, PI3K-AKT signaling, and metabolic pathways. These results are based on computational target prediction algorithms and therefore should be interpreted as hypothesis-generating rather than confirmatory evidence [42].

### 4.4. Clinical implications

This study provides evidence that a 20-miRNA signature may have utility for pancreatic cancer diagnosis. The signature’s performance in tissue samples (AUC 0.80) suggests applicability to biopsy specimens obtained via endoscopic ultrasound-guided fine-needle aspiration, while performance in serum samples (AUC 0.82) may support future investigation as a minimally invasive biomarker candidate [43]. We envision this signature as complementary to—rather than replacement for—existing diagnostic modalities including imaging and CA19−9. Future clinical implementation could involve multi-marker panels integrating miRNA signatures, protein biomarkers, and imaging features to maximize diagnostic accuracy.

The interpretability provided by SHAP analysis enhances clinical acceptability by identifying which miRNAs drive individual predictions, addressing “black box” concerns common to machine learning models in medicine [17]. However, as discussed in Section 4.7, prospective validation in well-characterized clinical cohorts with standardized protocols is essential before clinical implementation.

### 4.5. Comparison with prior studies

Several prior studies have developed miRNA signatures for pancreatic cancer diagnosis [11,44,45], with reported AUCs ranging from 0.75-0.90. Our study contributes through: (1) multi-cohort external validation (n = 767) across three independent datasets; (2) cross-platform assessment (microarray and RNA-seq); (3) transparent feature selection pipeline; and (4) explainable AI integration. The combination of multi-cohort validation, cross-platform consistency, and transparent methodology strengthens confidence in the signature’s robustness.

### 4.6. Strengths

Key strengths include: (1) multi-cohort validation design (n = 767 across training and three validation datasets); (2) transparent and training-restricted feature selection with complete parameter documentation and training-only selection; (3) explainable AI integration for biological interpretability; (4) cross-platform consistency across microarray and RNA-seq technologies; (5) stratified analysis by sample type demonstrating robustness; and (6) rigorous batch correction combined with stratified validation.

### 4.7. Limitations and future direction

Several important limitations warrant consideration. First, this study analyzed publicly available retrospective datasets, which limit control over clinical variables, sample collection protocols, and patient selection criteria. Prospective validation in independent clinical cohorts with standardized protocols is essential to confirm diagnostic performance in real-world settings and enable direct CA19−9 comparison.

Second, combining serum (n = 182) and tissue (n = 585) samples introduces biological compartment differences. Although ComBat normalization was applied and stratified validation demonstrated broadly consistent performance across sample types, biological compartment effects cannot be fully eliminated through statistical correction alone and represent an inherent limitation of multi-platform integration. Future studies focusing on single biological compartments with larger sample sizes may further refine compartment-specific performance estimates.

Third, pathway enrichment analyses relied on computational predictions rather than experimental validation. Experimental confirmation through luciferase assays, AGO2-CLIP, or proteomic profiling is needed to establish mechanistic roles.

Fourth, approximately 30% of signature miRNAs (6/20), including a data-driven contributor miR-6875-5p, have limited prior evidence in pancreatic cancer. Mechanistic validation studies are needed to elucidate their biological roles.

Fifth, clinical annotation heterogeneity across public datasets prevented detailed subgroup analyses by stage, grade, or molecular subtypes. Early-stage (I/II) representation was limited in some cohorts, affecting assessment of early detection utility.

Finally, validation cohorts predominantly represent Western populations. Independent validation in ethnically diverse cohorts is needed for global generalizability.

Future research priorities include: (1) prospective validation with standardized protocols and direct CA19−9 comparison; (2) experimental mechanistic validation of novel miRNAs through functional assays and AGO2-CLIP [REF: Hafner 2010]; (3) early-stage enriched cohorts for screening assessment; (4) evaluation of combination strategies integrating miRNA with CA19−9, ctDNA, and imaging biomarkers; (5) longitudinal studies for risk stratification; and (6) ethnically diverse validation cohorts.

## 5. Conclusion

This study developed and externally validated an interpretable 20-miRNA signature for pancreatic cancer diagnosis using a Random Forest framework. The model demonstrated consistent performance across independent cohorts (n = 767; cross-validation AUC 0.87; external validation AUC 0.78–0.83; sensitivity 84.7%; specificity 83.1%) with cross-platform robustness.

Although based on retrospective public datasets and lacking direct clinical comparator testing, the structured validation strategy, training-only feature selection, and explainable modeling approach provide methodological transparency and support acceptable generalizability within the studied cohorts.

Prospective validation in well-characterized clinical populations with standardized sample collection will be required before clinical translation. With further confirmation, this miRNA signature may represent a complementary diagnostic approach for early detection of pancreatic cancer.

## Supporting information

S1 Table Cross-Platform Performance Comparison. (XLSX)

S2 Table Complete miRNA Feature List with Annotations. (XLSX)
